# Toward Developmental Connectomics of the Human Brain

**DOI:** 10.3389/fnana.2016.00025

**Published:** 2016-03-31

**Authors:** Miao Cao, Hao Huang, Yun Peng, Qi Dong, Yong He

**Affiliations:** ^1^State Key Laboratory of Cognitive Neuroscience and Learning and International Data Group/McGovern Institute for Brain Research, Beijing Normal UniversityBeijing, China; ^2^Department of Radiology, Children's Hospital of PhiladelphiaPhiladelphia, PA, USA; ^3^Department of Radiology, Perelman School of Medicine, University of PennsylvaniaPhiladelphia, PA, USA; ^4^Department of Radiology, Beijing Children's Hospital Affiliated to Capital Medical UniversityBeijing, China

**Keywords:** connectomics, network, hub, rich club, brain development, ADHD, autism, dyslexia

## Abstract

Imaging connectomics based on graph theory has become an effective and unique methodological framework for studying structural and functional connectivity patterns of the developing brain. Normal brain development is characterized by continuous and significant network evolution throughout infancy, childhood, and adolescence, following specific maturational patterns. Disruption of these normal changes is associated with neuropsychiatric developmental disorders, such as autism spectrum disorders or attention-deficit hyperactivity disorder. In this review, we focused on the recent progresses regarding typical and atypical development of human brain networks from birth to early adulthood, using a connectomic approach. Specifically, by the time of birth, structural networks already exhibit adult-like organization, with global efficient small-world and modular structures, as well as hub regions and rich-clubs acting as communication backbones. During development, the structure networks are fine-tuned, with increased global integration and robustness and decreased local segregation, as well as the strengthening of the hubs. In parallel, functional networks undergo more dramatic changes during maturation, with both increased integration and segregation during development, as brain hubs shift from primary regions to high order functioning regions, and the organization of modules transitions from a local anatomical emphasis to a more distributed architecture. These findings suggest that structural networks develop earlier than functional networks; meanwhile functional networks demonstrate more dramatic maturational changes with the evolution of structural networks serving as the anatomical backbone. In this review, we also highlighted topologically disorganized characteristics in structural and functional brain networks in several major developmental neuropsychiatric disorders (e.g., autism spectrum disorders, attention-deficit hyperactivity disorder and developmental dyslexia). Collectively, we showed that delineation of the brain network from a connectomics perspective offers a unique and refreshing view of both normal development and neuropsychiatric disorders.

## Introduction

Brain development is characterized by complicated microstructural and macrostructural processes that span from the appearance of the first neurons to the establishment of the fully functioning adult brain. Revealing these complicated processes is important to understanding the formation of neural circuits and brain functions. Previous developmental hypotheses were mostly summarized from behavior or neuron perspectives, such as “Hebbian learning” (Hebb, 1949) or the “orthogenetic principle” (Werner, 1957; Sameroff, 2010), which are still in need of neurobiological evidence. With the recent advancement of non-invasive neuroimaging techniques and their applications to the pediatric population, comprehensive macroscopic brain structure and activity can be readily accessed in children *in vivo*. Studies employing advanced imaging techniques have revealed that regional structure and function develop according to specific principles, with a well-known example being that the regions responsible for higher-level cognition are the last to fully mature (Tau and Peterson, 2010; Dennis and Thompson, 2013a; Dehaene-Lambertz and Spelke, 2015).

Imaging connectomics, which evaluates the inter-regional structural and functional connectivity patterns among regions, has opened new avenues toward understanding the organization and function of the human brain (Sporns et al., 2005; Biswal et al., 2010; Sporns, 2011). The brain is believed to support global and local information communication through an integrative network (Bullmore and Sporns, 2009, 2012). With the establishment of the NIH Human Connectome Project, the importance of describing the network and its development trajectory was recently underscored (Van Essen et al., 2013). Using graph theory, recent studies on connectomics have demonstrated a number of nontrivial topological features in adult human brain networks, including their efficient small-world architecture, prominent modular structure, and highly connected and centralized network hubs (He and Evans, 2010; Stam, 2010; Bullmore and Bassett, 2011; van den Heuvel and Sporns, 2013; Berchicci et al., 2015). These brain network properties have been observed to be established as early as birth and exhibit continuous and dramatic maturational changes throughout infancy, childhood and even adolescence (Power et al., 2010; Collin and van den Heuvel, 2013; Dennis and Thompson, 2013b; Menon, 2013; Vertes and Bullmore, 2014).

With a collection of publications on the structural and functional network development, several questions emerge. Do the structural and functional brain networks develop with different maturation patterns? Are the developmental patterns different across age-ranges, such as during infancy and childhood? Do developmental brain disorders exhibit an abnormal developmental profile in brain networks compared with normal populations? In this review, we aimed to shed light on these important questions by collecting information regarding the recent progress in research on typical and atypical development of human brain networks from birth to early adulthood, focusing specifically on studies using advanced neuroimaging techniques and graph theoretical approaches. First, we introduce basic concepts about imaging connectomics, with a particular emphasis on graph-based network analysis approaches. Second, we discuss the recent findings on the healthy development of brain connectomes with different imaging modalities, concerning the developmental changes of topological properties. Third, we briefly mention abnormal network development in neuropsychiatric disorders [e.g., attention-deficit hyperactivity disorder (ADHD), autism spectrum disorder (ASD), and developmental dyslexia]. Finally, we discuss the limitations and future considerations of brain network development using imaging connectomics approaches.

## Brain connectome and graph theory

### Brain connectome construction

In graph theory, a network can mathematically be modeled as a graph with a set of discrete elements (nodes or vertices) and their mutual relationships (edges or links), which can be summarized in the form of a connection matrix. In the context of brain networks, nodes usually represent imaging voxels, regions of interest, or sensors, whereas links represent structural, morphological or functional connections, depending on the imaging modality considered (Bullmore and Sporns, 2009, 2012; He and Evans, 2010). In particular, structural connectivity can be obtained by reconstructing diffusion MRI (dMRI)-traced white matter projections (Mori and van Zijl, 2002; Hagmann et al., 2007; Gong et al., 2009) or through computing the covariance of brain morphological features among regions (e.g., gray matter volume or cortical thickness) derived from structural MRI (sMRI) data (Lerch et al., 2006; He et al., 2007). Functional connectivity can be measured by examining synchronous neural activity over the distributed brain areas with functional MRI (fMRI), electroencephalography/magnetoencephalography (EEG/MEG), or functional near-infrared spectroscopy (fNIRS; Friston, 1994; Micheloyannis et al., 2006; Niu and He, 2014). Once network nodes and connections are defined, a brain network can be obtained and further classified as directed or undirected, based on whether the edges have a sense of direction, and as unweighted (binary) or weighted, based on whether the edges in the graph have strength information. The present review focuses on the undirected binary or weighted brain networks. Notably, to avoid confusion, we used structural connectivity networks to refer to those constructed with white matter tracts and structural covariance networks for the morphological covariance based ones. Below, we briefly introduce several key graph theory metrics for network descriptions. For more details, see (Rubinov and Sporns, 2010; Stam, 2010; Bullmore and Bassett, 2011).

### Segregated and integrated network measures

**Figure 1:**
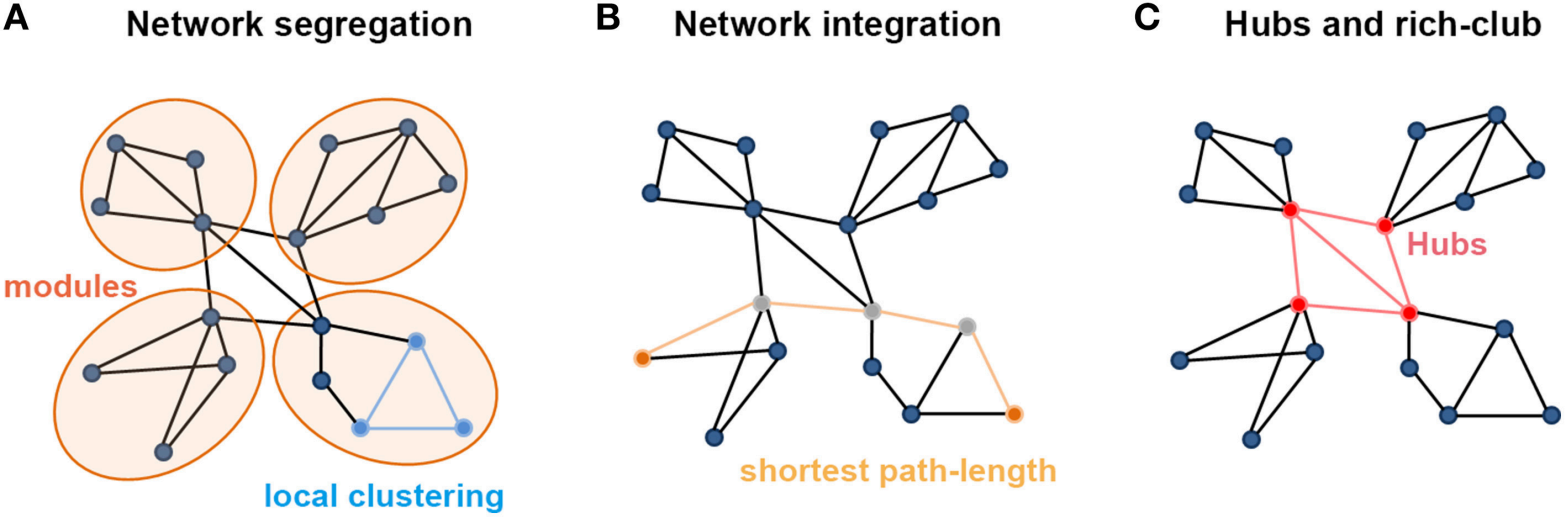
**Summary of the main measures with graph theoretical analysis. (A)** Metrics regarding the segregation of a network. Local clustering describes the tendency of nodes to form local triangles, providing insight into the local organization of the network. There are four modules in the graph in which connections within modules are much denser than connections between them. **(B)** Metrics about the integration of a network. The shortest path length describes the minimum number of steps needed to travel between two nodes (dots in yellow) and provides insight into the capacity of the network to communicate between remote regions. **(C)** The existence of a small set of high-degree nodes with a central position in the network may suggest the existence of hub nodes. High-level connectivity (lines in red) between hub nodes (dots in red) may suggest the existence of a central so-called rich club within the overall network structure.

Segregation and integration represent crucial information processing patterns of the brain, which ensure functional specialization and efficient global communication (Rubinov and Sporns, 2010; Sporns, 2013). Specifically, topological segregation (or local clustering) in the brain's information processing refers to the neuronal processing carried out among groups of regions or within modules (i.e., sets of nodes that are highly inter-connected but with relatively fewer connections to the others in different modules; Figure 1A). Clustering coefficients and modularity are two related metrics that quantify the features of topological segregation in brain networks. Mathematically, the clustering coefficient is defined by the fraction of the node's neighbors that are also neighbors of each other (Watts and Strogatz, 1998), while the modularity is determined by a single statistic of reflecting the modular structures of a network (Newman, 2006; Blondel et al., 2008). By contrast, integration refers to the efficiency of global information communication or the ability to integrate distributed information in the network, which is usually measured by the characteristic path length of a network, i.e., the average shortest path length between nodes (Figure 1B; Watts and Strogatz, 1998). Here, a path is a unique sequence of edges that connects two nodes with each other, and its length is given by the number of steps (in a binary graph) or the sum of the edge lengths (in a weighted graph), with the shortest one referred to as the shortest path length. Notably, in a complementary form, Latora and Marchiori (2001) also defined the local efficiency of each node, which is similar but not equivalent to its clustering coefficient or fault tolerance, and global efficiency that is inversely proportional to the characteristic path length of the network, thus allowing computation of a finite value for graphs with disconnected nodes.

Based on perspectives of information segregation and integration, networks can be divided into different types, including regular, small-world and random networks. Notably, a small-world structure characterizes an optimized balance between segregation and integration, which is essential for high synchronizability and fast information transmission in a complex network (Watts and Strogatz, 1998; Latora and Marchiori, 2001). A small-world network has both high global and local information transformation capacity, which is characterized as a shorter characteristic path length than a regular network and a greater clustering coefficient than a random network. Quantitatively, a small-world network is examined with the measurements of the normalized characteristic path length, defined as the ratio of the characteristic path length of the brain network to that of matched random networks, and the normalized clustering coefficient, defined as the ratio of the clustering coefficient of the network to that of matched random networks (Watts and Strogatz, 1998). Typically, for small-world networks, the ratio between the normalized characteristic path length and the normalized clustering coefficient should be >>1 (Humphries and Prescott, 2005; Achard et al., 2006).

### Hubs and rich-clubs

In brain networks, nodal regions that are positioned to make strong contributions to global network communication can be identified as network hubs using numerous different graph measures (van den Heuvel and Sporns, 2013). The simplest graph measure used for identifying hubs is degree centrality, which evaluates the number of connections attached to a given node (Figure 1C). Another measurement is betweenness centrality, defined as how many of the shortest paths between all other node pairs in the network pass through a given node, which reflects the ability of information transformation (Freedman, 1977). Nodal efficiency is also a frequently used measurement, which scales the average shortest path length between the given node and all the other nodes in the network (Achard and Bullmore, 2007). Importantly, these high-degree or high-central hubs strongly tend to be densely interconnected and form a rich-club structure in the brain organization (Figure 1C; van den Heuvel and Sporns, 2011). These hubs and rich-clubs are found to play important roles in global information transformation at the expense of relatively higher wiring, running costs, and vulnerability (Bullmore and Sporns, 2012; van den Heuvel et al., 2012; Liang et al., 2013; Tomasi et al., 2013).

## Typical development of healthy brain connectomes

Here, we focused on the development of the human brain connectome during the first two decades of life, in which dramatic brain structure changes happen and complex cognitive functions emerge (Giedd and Rapoport, 2010; Tau and Peterson, 2010). By searching PubMed (http://www.ncbi.nlm.nih.gov/pubmed) using the keywords “graph theory,” “small world,” “connectome” and “development” or “maturation,” we selected articles that used graph theory to analyze the brain networks based on MRI, fNIRS and EEG/MEG data. In total, we included 43 papers and discussed the development patterns of topological properties of brain connectomes (Tables 1, 2). According to the literature we reviewed, we found that the development of structural and functional brain connectomes followed distinct changing patterns from infancy to childhood and adolescence periods. Thus, we separately discussed the brain's structural and functional development, with each section proceeding chronologically from infancy (approximately 0–2 years old) to childhood and adolescence (approximately 2–10 years old).

**Table 1:** **Overview of studies about structural network development**.

	**Study**	**Modality**	**Subject n: ages**	**Network type**	**Node numbers**	**Connectivity metrics**
Infancy	Yap et al., 2011	DTI	39 sub (longitudinal): 2 wk, 1 y, 2 y	S B	78 (AAL template)	Deterministic tractography
	Tymofiyeva et al., 2012	DTI	17 sub: 6 mo	S B	40	Deterministic tractography
	Tymofiyeva et al., 2012	DTI	8 sub: 31.14-39.71 wk 8 sub: 1–14 d; 10 sub: 181–211 d; 7 sub: 24–31 y	S B	100	Deterministic tractography
	Ball et al., 2014	DTI	28 infants: 25–33 PMA 63 infants: 38–44 PMA	S B	100	Deterministic tractography
	van den Heuvel et al., 2015	DWI fMRI	27 infants: 27/1.6 PMA 27 infants: 30.8/0.7 PMA 42 adults: 29/8.0 y	S W	56	Deterministic tractography; Pearson's correlation
	Fan et al., 2011	sMRI	28 infants (longitudinal): 6.1 ± 2.8 wk, 59.3 ± 3.0 wk, 100.7 ± 6.8 wk; 27 adult controls: 24 ± 3 y	S B	90 (AAL template)	Pearson's correlation of the regional gray matter volume
Childhood and adolescence	Hagmann et al., 2010	DTI fMRI	30 sub for anatomical networks: 18 mo–18 y; 14 sub for functional networks: 2–18 y	S W S W	66 241	Deterministic tractography; Pearson's correlation
	Echtermeyer et al., 2011	DTI	9 sub: 12–14 y; 20 sub: 15–17 y; 16 sub: 18–20 y; 8 sub: 21–23 y	S W	414, 813, 1615	Deterministic tractography
	Dennis et al., 2013a	HARDI	47 sub: 12.3 ± 0.18 y; 55 sub: 16.2 ± 0.37 y; 336 sub: 23.6 ± 2.2 y	S B	68	Deterministic tractography
	Dennis et al., 2013b	HARDI	439 sub: 12–30 y	S W	70 (Desikan–Killiany atlas)	Deterministic tractography
	Chen et al., 2013	DTI	36 sub: 6.0–9.7 y 36 sub: 9.8–12.7 y; 36 sub: 12.9–17.5 y; 36 sub: 17.6–21.8 y; 36 sub: 21.9–29.6 y	S W	78 (AAL template)	Deterministic tractography
	Grayson et al., 2014	HARDI fMRI	15 sub: 7–11 y; 14 sub: 24–35 y	S W S W	219	Deterministic tractography; Pearson's correlation
	Lim et al., 2015	DTI	121 sub: 4–40 y	S W	82 (Freesurfer parcellation)	Deterministic tractography
	Huang et al., 2015	DTI	25 neotates: 37–43 wk; 13 toddlers: 1.79–3.12 y; 25 preadolescents: 10.7–13.5 y; 18 adults: 25–44 y	S W	80 (AAL template)	Probabilistic tractography
	Zhao et al., 2015	DTI	113 sub: 9–85 y	S W	1024	Deterministic tractography
	Baker et al., 2015	HARDI	31 sub (longitudinal): 15.58–17.94 y, 17.89–19.96 y	S W	80 (Freesurfer parcellation)	Probabilistic tractography;
	Koenis et al., 2015	DTI	183 sub (longitudinal): 9.9 ± 1.4 y; 12.9 ± 1.4 y	S W	90 (AAL template)	Deterministic tractography
	Wierenga et al., 2015	DTI	85 sub: 7.0–22.6 y; 38 sub: 7.4–22.9 y	S W	68 (Desikan–Killiany template)	Deterministic tractography
	Khundrakpam et al., 2013	sMRI	51 sub: 8.5–11.3 y; 51 sub: 11.4–14.7 y; 51 sub: 14.8–18.3 y	S B	78 (AAL template)	Pearson's correlation of the regional cortical thickness
	Alexander-Bloch et al., 2013	sMRI fMRI	108 sub for anatomical network: 11.1–20.0 y;	S W	360	Pearson's correlation of the regional cortical thickness and
			108 sub (longitudianl) for maturaltional network:	S W		the change rate of regional cortical thickness; Wavelet correlation
			9.0–22.8 y; 32 sub for functional network: 15.21–33.7 y	S W		
	Nie et al., 2013	sMRI	445 sub (longitudinal):3–20 y	S B	78 (AAL template)	Pearson's correlation of the regional cortical thickness and cortical folding

Sub, subjects; d, days; wk, weeks; mo, months; y, years; PMA, postmenstrual age; S, symmetric; W, weighted; B, binary; AAL, automatic anatomical labeling.

**Table 2:** **Overview of studies about functional network development**.

	**Study**	**Modality**	**Subject n: ages**	**Network type**	**Node numbers**	**Connectivity metrics**
Infancy	Fransson et al., 2011	fMRI	18 infants: 39 wk and 2 days; 18 sub: 22–41 y	S B	Voxel-wise	Pearson's correlation
	Gao et al., 2011	fMRI	51 neonates: 23 ± 12 d; 50 sub: 13 ± 1 mo; 46 sub:24 ± 1 mo	S B	90 (AAL template)	Pearson's correlation
	Gao et al., 2014	fMRI	178 sub: 1mo; 132 sub: 12 mo; 100 sub: 24 mo	S W	Voxel-wise	Pearson's correlation
	Pruett et al., 2015	fMRI	64 sub: 6 mo; 64 sub:12 mo	S W	230	Pearson's correlation
	Homae et al., 2010	fNIRS	15 sub: 2–11 d; 21 sub: 102–123 d; 16 sub: 180–206 d	S W	47	Pearson's correlation
Childhood and adolescence	Fair et al., 2007	fMRI	49 sub: 7–9 y; 43 sub: 10–15 y; 47 sub: 21–31 y	S W	39	Pearson's correlation
	Fair et al., 2009	fMRI	66 sub: 7–9 y; 53 sub: 10–15 y; 91 sub: 19–31 y	S WB	34	Pearson's correlation
	Supekar et al., 2009	fMRI	23 sub: 7–9 y; 22 sub: 19–22 y	S WB	90 (AAL template)	Wavelet correlation
	Dosenbach et al., 2010	fMRI	238 sub: 7–30 y	S W	160	Pearson's correlation
	Uddin et al., 2011	fMRI DTI	23 children: 7–9 y; 22 adults: 19–22 y	S W D W S W	9	Partial correlation; Granger causality analyses; Diffusion MRI deterministic tractography
	Zuo et al., 2012	fMRI	1003 sub: ~15–40 y	S W	Voxel-wise	Pearson's correlation
	Wang et al., 2012	fMRI	137 sub: 8–79 y	S W	116 (AAL template)	Pearson's correlation
	Hwang et al., 2013	fMRI	28 children: 10–12 y; 41 adolescents: 13–17 y; 30 adults: 18–20 y	S BW	Voxel-wise, 160 (Dosenbach)	Pearson's correlation
	Wu et al., 2013	fMRI	60 sub: 5.7–18.4 y	S B	90 (AAL template)	Pearson's correlation
	Cao et al., 2014b	fMRI	126 sub: 7–85 y	S W	1024, 106 (Dosenbach), 131 (Yeo)	Pearson's correlation
	Betzel et al., 2014	fMRI DTI	126 sub: 7–85 y	S W S W	114 (Yeo)	Pearson's correlation; Diffusion MRI deterministic tractography
	Sato et al., 2014	fMRI	447 sub: 7–15 y	S W	325 (AT325 atlas)	Pearson's correlation
	Sato et al., 2015	fMRI	447 sub: 7–15 y	S W	28	Pearson's correlation
	Qin et al., 2015	fMRI	183 sub: 7–30 y	S W	116 (AAL template)	Pearson's correlation
	Gu et al., 2015a	fMRI	780 sub: 8–22 y	S W	264 (Power)	Wavelet correlation
	Boersma et al., 2011	EEG	227 sub: 5–7 y	S W	14	Synchronization likelihood
	Miskovic et al., 2015	EEG	61 sub: 7 y; 53 sub: 8 y; 52 sub: 9 y; 56 sub: 10 y; 47 sub: 11 y	S W	33	Phase lag index

Sub: subjects; d: days; wk: weeks; mo: months; y: years; S: symmetric; D: directed; W: weighted; B: binary; AAL: automatic anatomical labeling.

### Development of structural brain connectomes

#### Structural connectivity networks

Recent advances in dMRI and tractography methods enable us to noninvasively study human brain structural networks. Specifically, through mapping the local diffusivity of water molecules in brain tissues, dMRI tractography allows us to map structural connectivity by traced white-matter fibers with deterministicor probabilistic tractography methods (Mori et al., 1999; Mori and van Zijl, 2002; Behrens et al., 2007). Whole-brain structural connectivity networks are then constructed by linking distinct regions with detected fiber tracts (Hagmann et al., 2007; Gong et al., 2009).

##### Infancy

**Figure 2:**
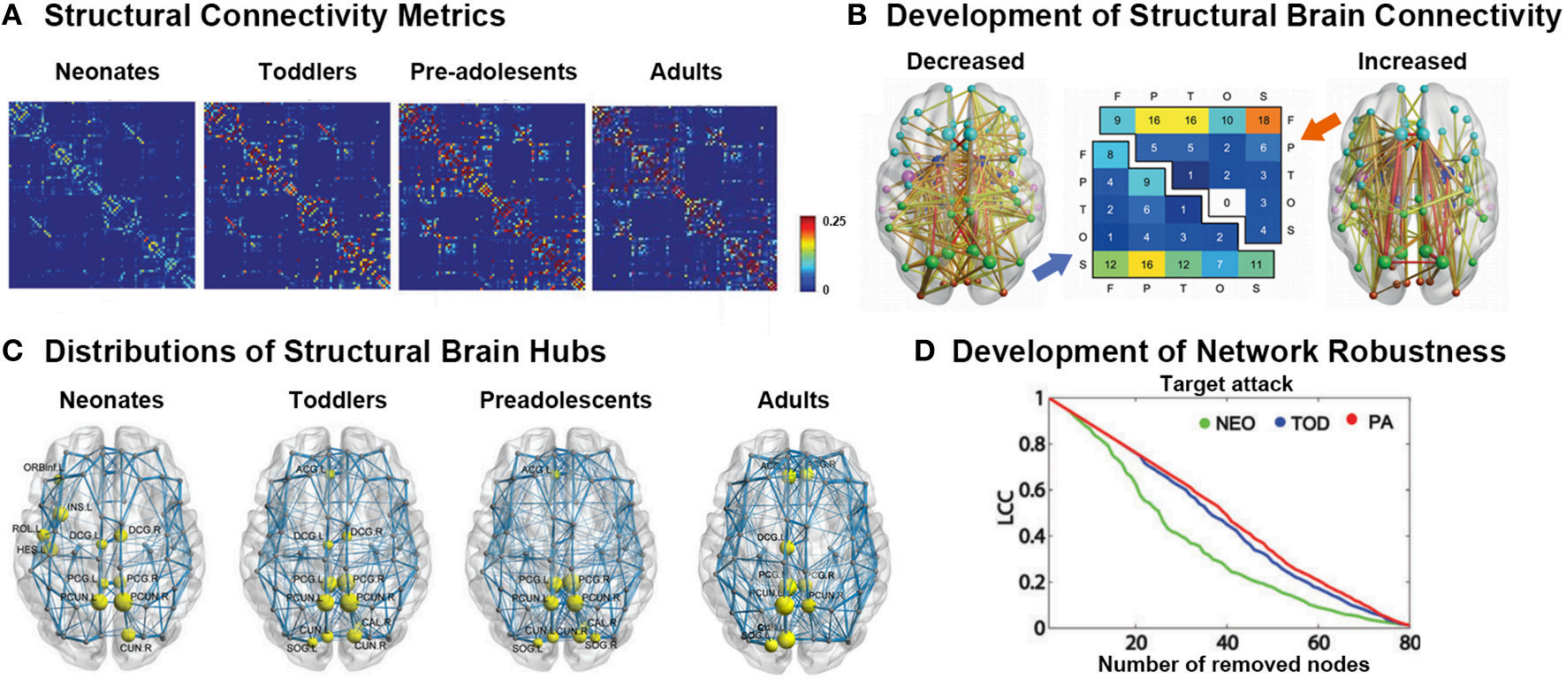
**Development of white-matter connectomes. (A)** Structural connectivity matrices of the neonates, toddlers, pre-adolescents, and adults group-averaged connectome. Adapted from Huang et al. (2015). **(B)** Late adolescent developmental changes in structural connectivity, with the thickness of each connection weighted by its associated one-tailed *t*-test statistic (FWE corrected, *p* < 0.05). Edge color represents connection type: non-hub to non-hub (yellow), hub to non-hub (orange), and hub to hub (red), with larger nodes corresponding to hub regions. Node color represents the assignment of each region of interest to one of five broad anatomical divisions: frontal (cyan), parietal (lime), temporal (magenta), occipital (orange-red), or subcortical (blue). The center panel illustrates the anatomical distribution of developmental decreases (lower triangular matrix) and increases (upper triangular matrix) in connectivity based on the classification of edges according to the anatomical divisions they interconnected. The values in these matrices represent relative proportions, calculated as the ratio between the frequency of edges linking each pair of divisions and the total number of edges belonging to the two categories. Adapted from Baker et al. (2015). **(C)** Distributions of hub regions in different age groups based on nodal efficiency centrality. PCG, precentral gyrus; PCUN, precuneus; CUN, cuneus; DCG, dorsal cingulate gyrus; INS, insular; ACG, anterior cingulate gyrus; SOG, superior occipital gyrus; ORBinf, inferior frontal gyrus; ROL, rolandic operculum; HES, Heschl's gyrus. Adapted from Huang et al. (2015). **(D)** Topological robustness of the structural networks in each group. The graphs show the AUC of the largest connected component (LCC) as a function of the removed node number by targeted attacks. The brain networks in the preadolescents (red line) were approximately as robust as those in toddlers (blue line) in response to both target failures. However, the neonates (green line) displayed remarkably reduced stability against both targeted attack and random failure compared with the other two groups. Adapted from Huang et al. (2015).

Using dMRI data, many studies have demonstrated that the adult-like topological organization of structural brain networks, such as the small-world, modular, hub, and rich-club structures, is well established by the time of birth (Figures 2A,C; Yap et al., 2011; Tymofiyeva et al., 2012, 2013; Ball et al., 2014; Huang et al., 2015; van den Heuvel et al., 2015). During the first few years of development, the topological structure of the brain structural connectivity networks were reported to exhibit increased global integration with decreased characteristic path length in approximately 6-month-old infants compared with term neonates (Tymofiyeva et al., 2013), increased global efficiency in 2-years-old toddlers compared with term neonates (Huang et al., 2015), as well as increased fiber length in 1-year-old infants compared with 2-week-old neonates (Yap et al., 2011). In contrast, decreased network segregation properties were reported, with a decreased clustering coefficient and modularity during the first half year (Tymofiyeva et al., 2013), as well as a decreased normalized clustering coefficient and modularity and increased number of modules and connectors in 2-year-old toddlers compared with term neonates (Huang et al., 2015). Moreover, although the degree distribution was found to follow a truncated power law across this period (Yap et al., 2011), which makes the network resilient to attacks, the network robustness to both random and targeted attack was reported to increase with age (Figure 2D; Huang et al., 2015), referring to the continuous refining of brain networks. Behavior al studies found that in half-year-old infants, the characteristic path length of brain structural networks inversely correlates with the neuromotor outcomes (Tymofiyeva et al., 2012).

Regionally, brain hubs were also found to be well-established by the time of birth (Figure 2C). Specifically, the hubs in neonates, calculated with both degree centrality and nodal efficiency, were found to be located in the medial superior parietal lobule and cuneus, which were adult-like, and in lateral regions including the rolandic operculum, Heschl's gyrus and sensorimotor regions, which were infant-specific (Gong et al., 2009; Yap et al., 2011; Huang et al., 2015; van den Heuvel et al., 2015). With development, the nodal efficiency of the medial hubs and fronto-medial regions was found to be significantly increased, whereas that of the regions located laterally decreased with age, until the hub locations in toddlers were highly similar to those in adults (Huang et al., 2015).

Gender differences in babies' brain networks were not detected until they were 2 years old, with females exhibiting higher global efficiency and lower local efficiency than males (Yap et al., 2011). Network asymmetry was already detected in neonates' brains, with an overall higher nodal betweenness in the right brain than the left brain, and this increased with age (Yap et al., 2011). Notably, this study reported increased local segregation and consistent global integration during early development, which was not consistent with the above-discussed papers (Tymofiyeva et al., 2013; Huang et al., 2015). Specifically, Yap et al. (2011) found that 2-week-old neonates' brain networks exhibit lower local efficiency but similar global efficiency compared with that of 1-year-olds and 2-year-olds, indicating the needs for further studies with larger sample sizes.

##### Childhood and adolescence

After the first few years, increased integration and decreased segregation were generally found to continue until adulthood (Hagmann et al., 2010; Chen et al., 2013; Dennis et al., 2013b; Huang et al., 2015). Specifically, from 2 years of age to adulthood, human brain structural networks experience the continued increases in global efficiency, nodal strength, number of modules and connectors and decreased local clustering and modularity (Hagmann et al., 2010; Uddin et al., 2011; Chen et al., 2013; Dennis et al., 2013b; Huang et al., 2015; Koenis et al., 2015; Wierenga et al., 2015; Zhao et al., 2015). The numbers of streamlines of fiber tracts, which were short, within modules and within hemispheres, were found to significantly decrease with development (Lim et al., 2015). Moreover, these types of topological changes were found to be highly heritable and significantly correlated with IQ (Koenis et al., 2015).

The location of hubs was found to be relatively consistent across this period, with subtle changes taking places (Figure 2C; Hagmann et al., 2010; Chen et al., 2013; Huang et al., 2015). Specifically, relatively strong developmental changes in the intra-lobe connections within the frontal and parietal lobes compared to changes in the temporal and occipital lobes and between subcortical structures were observed (Wierenga et al., 2015). Furthermore, the regions located within the default mode network were found to mature later than other regions (Chen et al., 2013; Zhao et al., 2015). The rich-club organization, which consisted of densely interconnected hubs and comprised the postero-medial core with extensions into the temporo-parietal junction and fronto-medial cortices, was also found to be established in the brains of children and remained stable with development (Hagmann et al., 2010; Chen et al., 2013; Dennis et al., 2013a; Grayson et al., 2014), with subtle connection changes, including decreased correlation within the subcortical hub and increased connections between the frontal and temporal as well the frontal and subcortical hubs (Figure 2B; Dennis et al., 2013b; Baker et al., 2015). Network motifs, a specific connectivity pattern, were found to change across ages, but they were significantly affected by template resolution (Echtermeyer et al., 2011). Meanwhile, anatomical measurement of fiber length was found to significantly increase during development (Zhao et al., 2015), with a robust distribution relative to the spatial resolution (Echtermeyer et al., 2011).

Gender differences during this period were reported, which included the earlier streamline losses (Lim et al., 2015) and significantly higher small-worldness and normalized local clustering in females than in males (Dennis et al., 2013b). Brain asymmetry was also found, including inverse development curves between the left and right hemispheres with respect to global efficiency, local clustering, and modularity (Dennis et al., 2013b). Notably, there were also some inconsistent findings, which are mainly reflected in increased local efficiency during development (Chen et al., 2013; Koenis et al., 2015; Wierenga et al., 2015). Given that both decreased (Lim et al., 2015) and increased fiber streamline counts (Chen et al., 2013) and increased mean fractional anisotropy (Koenis et al., 2015), as well as decreased average apparent diffusion coefficients, diffusivity, and radial diffusivity, were found during development (Hagmann et al., 2010; Wierenga et al., 2015), we inferred that different weighting methods may explain these different results. In an analysis of dMRI data from the same group, Koenis et al. (2015) found that fractional anisotropy weighted networks showed increased local efficiency, whereas fiber number weighted networks showed decreased local efficiency with development.

Taken together, these findings indicate that structural connectivity networks already exhibit adult-like organization at the time of birth and then experience continued increased integration and robustness with development, indicating the refining of brain circuits. Throughout this period, hub locations were relatively consistent in the postero-medial core, with extensions into the temporo-parietal junction and fronto-medial cortices, with fine-tuning in the strengthening of the frontal and temporal hubs, as well as weakening of the subcortical hubs and lateral non-hub regions in the cortex. Notably, increased FA was found to be significantly correlated with the changes in network properties, indicating that the development of network structure is associated with microstructural modifications of white matter, such as synaptogenesis and synaptic and axonal pruning, as well as myelination (Tau and Peterson, 2010; Huang et al., 2015). However, discrepancies between different studies also exist, which may be due to differences in network construction approaches and limited sample sizes.

#### Structural covariance networks

Structural covariance networks are established based on coordinated variations in brain morphology (e.g., gray-matter volume and thickness), which are established by structural MRI, as measures of structural association between regions (Lerch et al., 2006; He et al., 2007).

##### Infancy

There is only one work conducted by Fan et al. (2011), exploring the structural covariance network development during infancy. They found that the economic small world and modular structure were also established in the structural covariance networks of 1-month-old infants. During early development, from 1 month to 3 years old, network integration consistently enhanced with increased global efficiency, whereas network segregation properties showed inverted U-shape curves, with the modularity and local efficiency of 2-year-olds being higher than those of the brain networks of younger and older participants.

##### Childhood and adolescence

Khundrakpam et al. (2013) explored the development of structural covariance networks from early childhood to adulthood. Complex topological structure changes were detected: from 4 years to 11 years, network integration continuously enhanced characterized by increased global efficiency and numbers of connectors, whereas segregation decreased, characterized by decreased local clustering; from 11 years to 15 years, contrasting development curves were found, including decreased global efficiency and numbers of connectors with increased local efficiency; thereafter, the networks became stable until adulthood. However, a longitudinal study of a large sample with 3- to 29-year-old subjects, which employed two correlation calculation methods, the correlation of cortical thickness and cortical curvedness, to construct the brain networks, reported inconsistent findings (Nie et al., 2013). Specifically, the global efficiencies of both types of networks were found to decrease from 3 years old to 7 years old and then increase until approximately 9 years old and then become stable. In contrast, the local efficiency increased from 3 to 7 years old and then decreased with age. The peak age for both developmental curves was ~7 years old, when brain cortical thickness reaches its highest value and cortical folding becomes stable.

Although the network reorganized during the developmental period, the location of hubs was relatively consistent comprising the medial posterior parietal and frontal core and some temporal regions with subtle changes from the language-related regions to the frontal lobes (Fan et al., 2011; Khundrakpam et al., 2013; Nie et al., 2013). Regional analysis also found that the primary regions matured earlier and were well developed by 5 years old, followed by the paralimbic and association regions, which developed mainly during early to late childhood (~5–11 years old; Khundrakpam et al., 2013).

Recently, one work employed the similarity in maturational curves of cortical thickness between regions in participants ranging from 6 to 12 years old to construct brain structural networks (Alexander-Bloch et al., 2013). They found that the topological properties of these maturational networks exhibit similar topological properties to the structural covariance networks. Furthermore, both the maturational and structural covariance networks could predict the functional networks well. These findings indicate that maturational trajectories may underlie the properties of structural covariance networks, as well as functional networks.

In summary, during development the global topological properties of structural covariance networks undergo complicated changes, which still need further exploration. In contrast, the regional findings were relatively consistent in the hub locations, which were similar to the hubs in structural connectivity networks. The developmental order from primary to high functioning regions was also detected. Notably, the structural covariance connections were previously reported to partly reflect underlying fiber connections but contain exclusive information (Gong et al., 2012). Specifically, graph theoretic analysis reveals that the thickness correlation network has a more randomized overall topology than the structural connectivity network, whereas the regional characteristics in these two networks are statistically correlated, which may be in agreement with the findings during development.

### Development of functional brain connectomes

The functional network in the human brain *in vivo* can be constructed from EEG/MEG, fNIRS, and fMRI data by calculating the temporal correlation between the fluctuations in measured electric, magnetic and blood oxygen level-dependent signal. Specifically, the resting-state functional imaging data measures the endogenous or spontaneous brain activity of subjects who are not performing any specific tasks, which is very suitable for the study of development (Biswal et al., 1995; Stam, 2010; Niu and He, 2013).

#### Infancy

Studies that employed resting-state fMRI (rsfMRI) have found that typical organizational principles, such as the existence of hubs and small-world structure, were already present by the time of birth (Fransson et al., 2011; Gao et al., 2011). In the first 2 years of life, both the functional network integration and segregation properties were found to significantly increase with age from birth to 1 year of age (Gao et al., 2011). Thereafter, the network efficiency became stable. The robustness of the networks linearly increased with age (Gao et al., 2011). Global and local efficiency in the specific functional network of the sensorimotor system significantly increased from 1 year of age to 2 years of age, which was also reported with MEG data (Berchicci et al., 2015).

**Figure 3:**
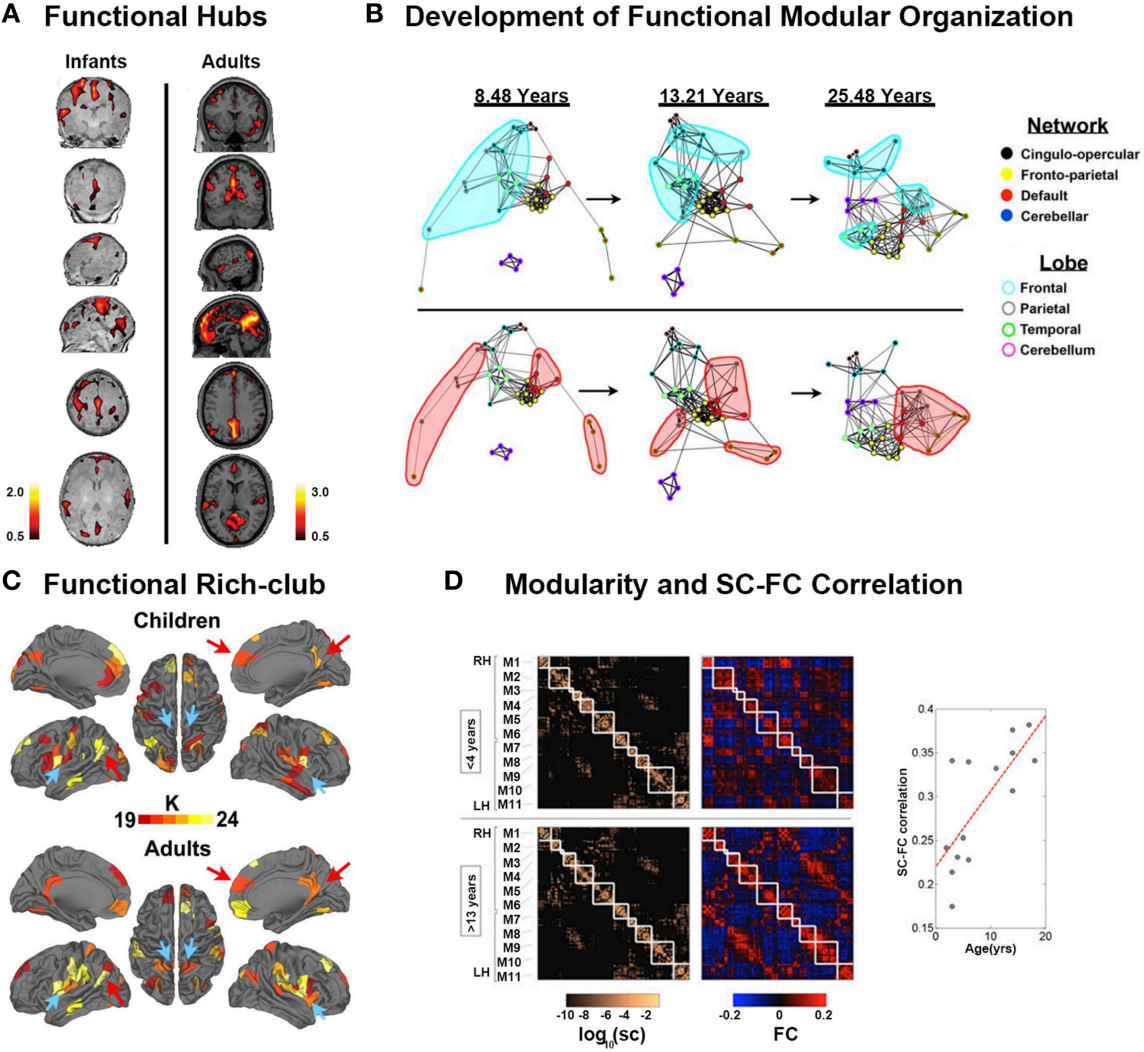
**Development of functional connectomes. (A)** Distribution of hub regions in the functional networks of infants and adults based on degree centrality. In infants, the majority of cortical hubs were located in the homomodal cortex, mostly in the auditory, visual, and sensorimotor areas, and to a lesser extent in the PFC. Prominent locations for hubs in adults included the precuneus/posterior cingulate cortex, medial PFC, anterior cingulate cortex, bilateral parietal lobule, and bilateral insula. Adapted from Fransson et al. (2011). **(B)** The figure showed the dynamic development of the default network, and cerebellar network using spring embedding. The figure highlights the segregation of local, anatomically clustered regions, and the integration of functional networks over development. Nodes are color coded by their adult network profile (core of the nodes) and by their anatomical location (node outlines). Connections with *r* > 0.1 were considered connected. Adapted from Fair et al. (2009). **(C)** The functional rich-club organizations in children and adults. Although many regions overlap (red arrows, for example), there are bilateral regions that appear only in adults (blue arrows, for example). Adapted from Grayson et al. (2014). **(D)** Modularity and SC–FC correlation. Cortical SC and FC matrices averaged over the younger (<4 years) and older (>13 years) age group. Structural modules are delineated by the superimposed white grid. Eleven modules (M1–M6 in the right hemisphere, M7–M11 in the left hemisphere) were identified, and the two sets of SC and FC matrices are displayed such that modules correspondence across age is maximized. Although modules are highly conserved (normalized mutual information = 0.82), there is a notable increase in SC–FC correspondence from younger to older brains. There is an increasing statistically significant relationship between SC and FC across age (*R* = 0.74, *p* < 0.005). Adapted from Hagmann et al. (2010).

Hub regions were also detected in newborn infants. Fransson et al. (2011) found that the functional hub regions in the brains of neonates born ~1 week before were located in primary regions, including sensorimotor cortex, caudate, supplementary motor area, superior temporal cortex, occipital cortex, and lateral and medial prefrontal cortex (Figure 3A). Gao et al. (2011) studied hub evolution during the early development. Specifically, they also detected that the regions located in the lateral frontal cortex, caudate, and occipital cortex acted as hubs in newborn neonates. With development, bilateral supplementary motor areas were noted among the hubs in 1-year-old infants. In 2-year-olds, the hub regions moved toward to areas involved in high order cognitive functions, such as the medial superior frontal gyrus (Gao et al., 2011). Notably, they found that the bilateral insula consistently performed as hubs for all three age groups. Moreover, during the first 2 years, the hub regions showed increases in their long-range connections to possess an increasingly more efficient strategy. Inter-subject variability was found to be relatively lower in primary functional areas but higher in association areas during the first 2 years (Gao et al., 2014). Although inter-subject variability in infants was similar to that in adults, specific patterns were still present in infants. Specifically, the medial prefrontal/anterior cingulated, auditory, subcortical and insula regions exhibited lower variability in infants than in adults, which may indicate “skill learning” development (Gao et al., 2014).

Consistent with increasingly efficient communication, connectional analysis found that during the first 6 months, the connections of the temporal, parietal and occipital cortex significantly increased with age, with the clusters comprising homolog regions formed (Homae et al., 2010). Meanwhile, the homotopic connections of the frontal regions decreased with age, whereas the connections of the prontoposterior regions decreased until ~3 months of age but then increased (Homae et al., 2010). Another study found that the thalamus-sensorimotor and thalamus-salience connectivities were found already formed in neonates, whereas the thalamus-medial visual and thalamus-default mode network connectivity emerged at 1 year of age (Alcauter et al., 2014). Moreover, classification analysis revealed that the functional connectivity could provide critical information to accurately identify infants at high-risk for autism versus infants at low-risk, both in 6-month-old and 12-month-old infants (Pruett et al., 2015).

#### Childhood and adolescence

After early development, brain functional networks still showed increased segregation with increased local clustering or local efficiency, within-module connectivity, and network hierarchy after 5 years of age (Supekar et al., 2009; Dosenbach et al., 2010; Boersma et al., 2011; Wu et al., 2013; Betzel et al., 2014; Cao et al., 2014b). Notably, increased global efficiency (Miskovic et al., 2015) and long distance connections (Fair et al., 2007, 2009; Supekar et al., 2009; Dosenbach et al., 2010; Cao et al., 2014b), as well as the organization of modules, shifted from a local anatomical emphasis in children to a more distributed architecture in young adults (Figure 3B; Fair et al., 2009), indicating an increased global integration process. Taken together, these findings indicated that the functional specification and integration in the brain increased during development. One recent study conducted a modular analysis of the subjects from 8 to 22 years old and found that different systems with diverse roles in whole-brain networks showed different change trajectories with development (Gu et al., 2015a). Specifically, sensorimotor systems and higher order cognitive systems (cognitive control, salience, memory, and attention systems), tending to be cohesive provincial and incohesive connector systems, respectively, all became increasingly segregated from other systems during development. Subcortical and cerebellar systems, tending to be incohesive provincial systems, became increasingly differentiated during development. Uniquely, the default mode system, tending to be a cohesive connector system, was shown to be both increasingly cohesive and increasingly associated with other systems during development.

Hub distributions after 5 years old were found to be stable until adulthood located at the insula, superior visual cortex, postcentral gyrus, thalamus, caudate, and default mode network (DMN), comprising the precunues/posterior cingulated cortex, angular cortex, superior frontal gyrus, parahippocampal, medial prefrontal cortex, and middle temporal gyrus (Zuo et al., 2012; Hwang et al., 2013; Wu et al., 2013; Cao et al., 2014b). Notably, these hubs intensely interconnected to form the rich-club organization. With development, the normalized rich-club coefficients, i.e., the connectivity between the hub regions, significantly increased (Figure 3C; Fair et al., 2008; Uddin et al., 2011; Cao et al., 2014b; Grayson et al., 2014), indicating enhanced communication between hubs. The regional properties of the frontal brain regions, superior temporal gyrus, and angular gyrus were found to increase with age, whereas those of the regions related to motor, somatosensory, auditory, and visual functions, as well as the bilateral precuneus and subcortical regions decreased with age (Supekar et al., 2009; Dosenbach et al., 2010; Wang et al., 2012; Zuo et al., 2012; Hwang et al., 2013; Wu et al., 2013; Cao et al., 2014b; Sato et al., 2014, 2015). These findings suggested that the regions for high order cognitive functions matured late compared with the primary regions. Moreover, the functional connectivity information could be used to accurately predict brain maturity (Dosenbach et al., 2010; Wang et al., 2012). Interestingly, recent neuroscience studies suggested that resting-state FC may be dynamic and exhibit significant spontaneous fluctuation (Kang et al., 2011; Hutchison et al., 2013). The spontaneous fluctuations of resting-state functional connectivity, which significantly increased with age, could be used to accurately predict brain age (Qin et al., 2015). Notably, the correlations between structural and functional connectivity showed an increasing trend with age (Figure 3D; Hagmann et al., 2010; van den Heuvel et al., 2015). In particular, the functional connectivity without direct structural connections was primarily strengthened with development (Betzel et al., 2014).

Gender effects explorations found that girls exhibited higher local clustering than boys (Boersma et al., 2011; Wu et al., 2013), whereas boys showed higher global efficiency than girls (Wu et al., 2013). Regional differences in gender were found in the DMN, language, sensorimotor, and the visual systems, which may indicate cognitive differences between females and males in visuospatial, language and emotion processing (Zuo et al., 2012; Wu et al., 2013). IQ was found to be significantly correlated with regional properties in the frontal, parietal and temporal lobes (Wu et al., 2013; Santarnecchi et al., 2014), which was consistent with the parieto-frontal integration theory of the integrative roles of these regions. However, inconsistent findings were also reported, including stable (Wu et al., 2013) or decreased global efficiency (Boersma et al., 2011; Cao et al., 2014b) and decreased modularity (Cao et al., 2014b; Miskovic et al., 2015) during development. Notably, functional networks were relatively sensitive to the choice of template, ways of computing correlations and methods for determining the threshold of the network (Wang et al., 2009a; Liang et al., 2012). All of these factors may account for the inconsistent findings. Further studies are still urgently needed to elucidate this problem.

In summary, the functional networks experienced more dramatic reorganization during development than the structural ones. Both increased information integration and segregation continuously progressed since birth. The hub locations were moved from the primary regions to those involving high-order cognitive functions as the organization of modules shifted from a local anatomical emphasis to a more distributed architecture. Moreover, the physiological bases including blood supply and glucose metabolism of functional network properties in both infants and adults and modulation in response to task demands were also detected (Chugani, 1998; Liang et al., 2013; Tomasi et al., 2013). Therefore, we inferred that functional networks matured with both the underlying structural networks and environment-driving training to meet cognitive challenges at different stages of life.

## Atypical development of brain connectomes in neuropsychiatric disorders

In this part, we briefly introduce the findings regarding abnormal brain networks in neurodevelopment disorders (ADHD, ASD and dyslexia) using imaging connectomics.

**Figure 4:**
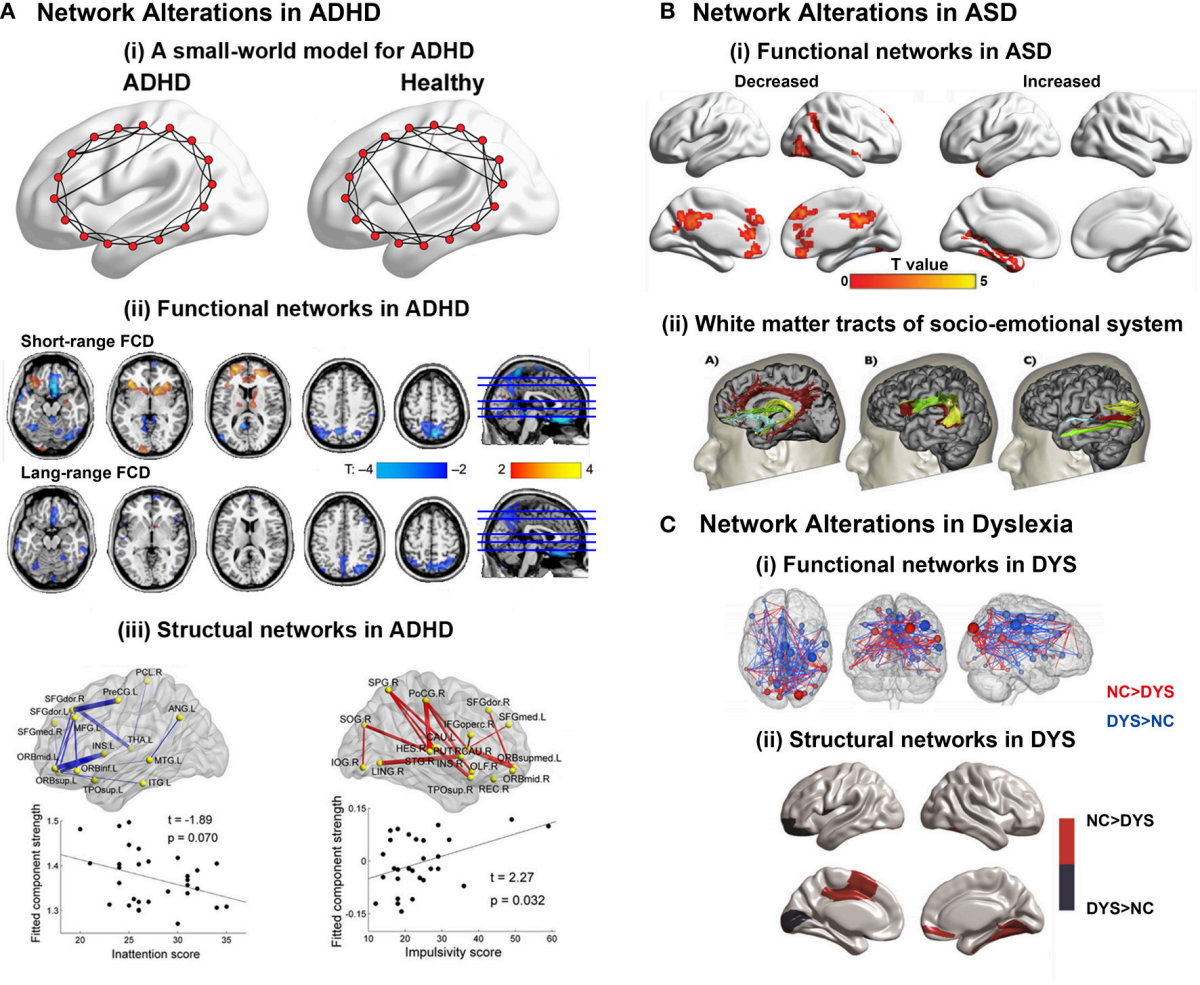
**(A)** Brain network alterations in ADHD. (i) Small-world models for ADHD and healthy brain networks. The ADHD networks showed a regular tendency compared with healthy controls. Adapted from Cao et al. (2014a). (ii) Decreased or increased functional connectivity density (FCD) in ADHD patients compared with healthy controls. Adapted from Tomasi and Volkow (2012). (iii) Decreased or increased white matter connections in ADHD participants compared with healthy controls and their relationships with the clinical characteristics of the patients. Blue curve: the significantly decreased network-based statistic (NBS) component; red curve: the significantly increased NBS component. Adapted from Cao et al. (2013). **(B)** Brain regions displaying disrupted functional connectivity in autism. (i) Voxels displaying altered functional connectivity in autism. Voxels that displayed weaker functional connectivity in the autistic population than in the controls are shown in blue, and the voxels that displayed stronger functional connectivity in the autistic population are shown in red. The color bar represents the degree of connectivity according to the number of significantly affected edges relating to a given voxel. Adapted from Eilam-Stock et al. (2014). (ii) White matter tracts of the socio-emotional processing system. Left: white matter tracts of the limbic system; middle: white matter tracts linking the mirror neuron system; right: white matter tracts of the face processing system. Adapted from Ameis and Catani (2015). **(C)** Brain network alterations in dyslexia. (i) Whole-brain functional connectivity differences between groups. Three-dimensional representation of healthy controls readers (NC) > dyslexic readers (DYS) and DYS > NC edge components (*p* < 0.01 after NBS correction). Adapted from Finn et al. (2014). (ii) Between-group differences in regional nodal characteristics in cortical thickness networks. Group differences of and nodal degree in cortical thickness networks. Blue represents the brain areas with significantly lower nodal properties in DYS than in NC, whereas red represents the brain areas with significantly higher nodal properties in NC than in DYS. Adapted from Qi et al. (2016).

ADHD is one of the most common neurodevelopment disorders in childhood, with core symptoms of inattention, hyperactivity and impulsivity (American Psychiatric Association. DSM-5 Task Force, 2013). Convergent evidence suggested that children with ADHD had abnormal small-world properties in both functional and structural brain networks characterized by higher local clustering and lower global integrity, indicating a disorder-related regular shift in organizational properties (Figure 4A; Wang et al., 2009b; Ahmadlou et al., 2012a,b; Cao et al., 2013). Regional and connectional alterations were found to be mainly involved in the default-mode, attention, sensorimotor, and subcortical systems (Figure 4A; Fair et al., 2010, 2013; Colby et al., 2012; Tomasi and Volkow, 2012; Di Martino et al., 2013; Sripada C. et al., 2014a). Specifically, the two primary symptoms of ADHD were found to be correlated with different connectivity changing patterns. Decreased connectivity in prefrontal-dominant circuitry and increased connectivity in orbitofrontal-striatal circuitry correlated with behavioral scores of inattention and hyperactivity/impulsivity symptoms, respectively (Figure 4A; Tomasi and Volkow, 2012; Cao et al., 2013; Fair et al., 2013). Notably, a developmental perspective has recently been increasingly noted in the research of psychiatric disorders (Di Martino et al., 2014). For ADHD, a delayed developmental model has been proposed (Fair et al., 2010; Cao et al., 2014a). Specific maturational lag in functional connections within the DMN and in DMN interconnections with the frontoparietal network and ventral attention network were detected (Sripada C. S. et al., 2014b). Further studies are still in needed to test the hypothesis from the connectomic perspective using both multimodality data and whole-brain topological analysis.

ASD manifests early in development and is characterized by deficits in social interaction and communication, as well as stereotyped and repetitive behaviors and restricted interests in domains of activities (American Psychiatric Association. DSM-5 Task Force, 2013). The findings about alterations in global topological architecture in ASD were inconsistent. Whereas the topological properties of functional networks were found to exhibit a randomized tendency of decreased segregation, both decreased and increased local clustering were found in the structural networks of ASD patients (Rudie et al., 2012; Jakab et al., 2013; Li et al., 2014; Valk et al., 2015). In contrast, the regional findings suggested the disruption of both functional and structural hubs in ASD (Figure 4B; Di Martino et al., 2013; Crossley et al., 2014; Eilam-Stock et al., 2014). Connectional analyses show hypoconnectivity in the so-called “social network” encompassing the default mode, attention and executive networks, and hyperconnectivity in limbic regions (Figure 4B; Anderson et al., 2011; Gotts et al., 2012; Rudie et al., 2012; Tyszka et al., 2014; Cheng et al., 2015). Specifically, a lack of long-range connections and an increase in short-range connections in ASD patients compared with healthy controls were reported (Khan et al., 2013; Bernhardt et al., 2014; Ameis and Catani, 2015; Kitzbichler et al., 2015). For ASD, an overgrowth developmental hypothesis has been raised (Courchesne et al., 2007; Uddin et al., 2013). Interestingly, a recent DTI study conducted on 2-year-old babies showed significantly disturbed local and global efficiency in high-risk infants diagnosed with ASD, compared with both low- and high-risk infants not diagnosed with ASD, indicating that the abnormality occurred at a very early stage (Lewis et al., 2014).

Developmental dyslexia, also known as reading disorder, is a neurobiological deficit characterized by persistent difficulty in learning to read in children and adults who otherwise possess normal intelligence (American Psychiatric Association. DSM-5 Task Force, 2013). Thus far, the connectomic studies in dyslexia are relatively few with divergent findings. Studies employing sMRI data to explore alterations in Chinese dyslexia found both decreased (Qi et al., 2016) and increased (Liu et al., 2015) local clustering with constant global efficiency in the structural networks compared with healthy participants. Moreover, structural networks of children with familial risk of reading difficulties showed no significant difference in global topological properties compared with healthy controls (Hosseini et al., 2013). Functional networks based on MEG data in dyslexia showed reduced global and local efficiency during both resting and task states compared with healthy controls (Vourkas et al., 2011; Dimitriadis et al., 2013). Regional alterations in both structural and functional networks were reported in the visual cortex for visual information integration and prefrontal areas for attention modulation, as well as the supramarginal gyrus, precentral gyrus, Heschil's gyrus, posterior cingulated, and hippocampus (Figure 4C; Hosseini et al., 2013; Finn et al., 2014; Liu et al., 2015; Valk et al., 2015; Qi et al., 2016). Interestingly, the hub locations in the structural networks of Chinese dyslexia were found to be more bilateral and anterior than those of healthy controls (Qi et al., 2016), which was consistent with the findings that in functional networks, non-impaired readers have stronger left lateralization for language than dyslexic readers, who rely on bilateral systems (Finn et al., 2014).

Together, many previous studies have shown topological disorganization of brain networks in these neurodevelopmental disorders. In the future, it will be important to compare commonalities and differences in developmental brain networks among these neuropsychiatric disorders. These imaging connectomics studies will be critical for deepening our understanding of developmental mechanisms and to discover biomarkers for early diagnosis, treatment evaluation, and identification of intervention targets.

## Conclusion and further considerations

**Figure 5:**
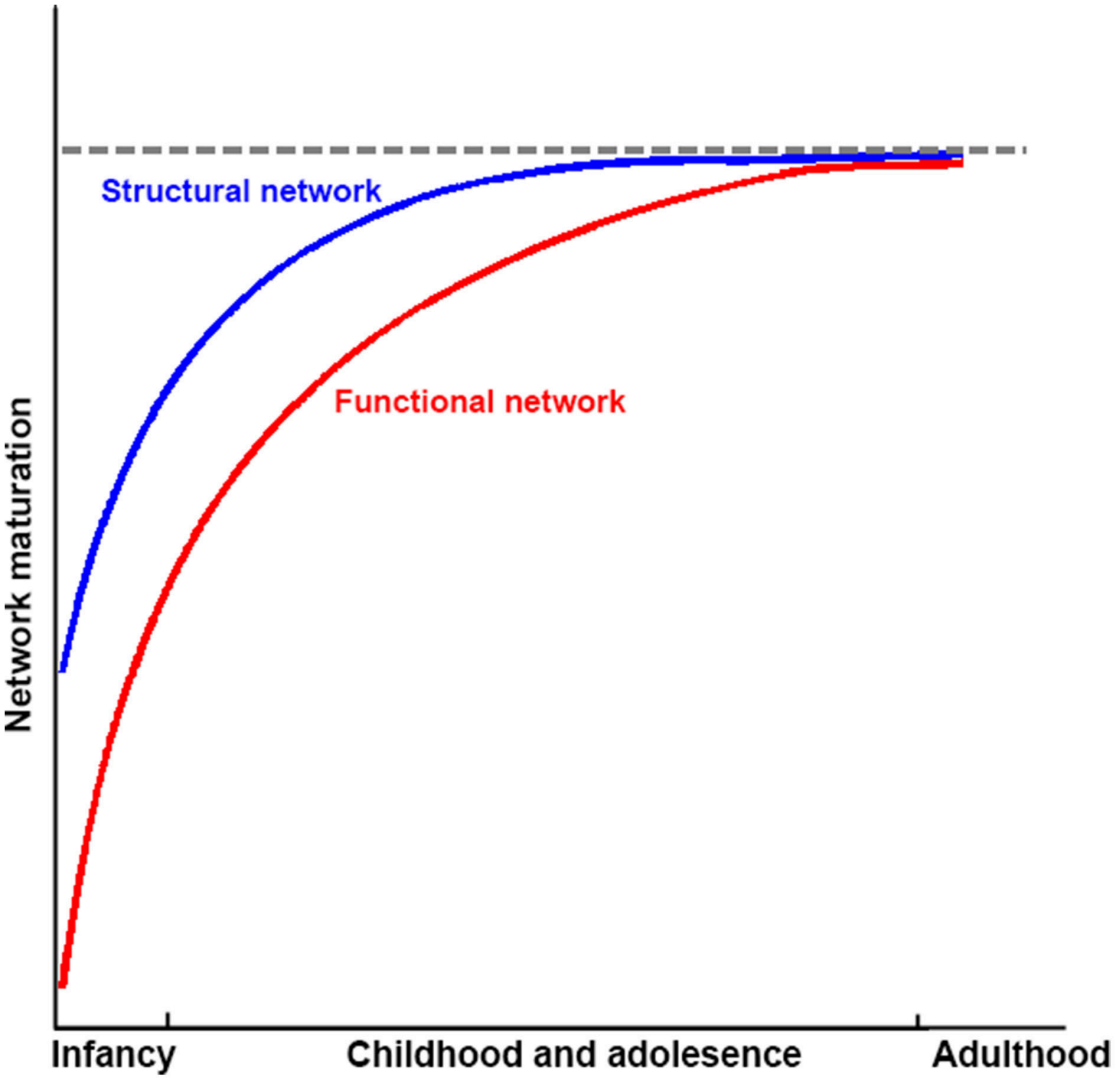
**Sketch plot showing the development of structural and functional brain networks in infancy and childhood and adolescence relative to healthy adults**.

Taken together, structural connectivity networks, structural covariance networks, and functional networks already exhibit an efficient small-world modular structure, with the appearance of hubs at the time of birth. The organizations of structural connectivity networks in infants were somewhat similar to those of adults, with the refining of enhanced network integration with development. In contrast, functional networks in infants showed dramatically different architecture from those in adults, although the critical topological structure was also established. With development, functional networks are reorganized, with both increased segregation and integration as hubs move from primary regions toward high order cognitive regions. These finding suggest that structural connectivity networks may mature earlier than the functional ones (Figure 5). Given that previous studies that employed empirical and simulated data have demonstrated that structural connectivity provides crucial structural substrates underlying the brain's functional connections in adults (Honey et al., 2009; van den Heuvel et al., 2009; Wang et al., 2015), this developmental pattern may reflect preparation for the potential structural constrains of further development of the brain's functional networks. Further studies are needed to verify this hypothesis. The brain networks constructed with structural covariance using sMRI data showed different maturational curves than those of either structural connectivity networks or functional networks. Specifically, structural covariance networks with sMRI are more complex and seem heavily affected by cortical morphological changes with development. Finally, the literature reviewed here suggests abnormal network development in populations with developmental psychiatric disorders, such as ADHD and ASD.

Although these findings shed light on our understanding of human development at a macroscopic level, some issues and problems still need to be addressed. First, most of the connectome developmental changes were detected based on cross-sectional data; thus, they may be influenced by inter-subject variability and unbalanced cohort distributions. Thus far, only a few studies regarding the structural connectome development have employed longitudinal data, with relatively small sample sizes. Investigations of longitudinal network dynamics with large sample sizes should be under taken in the future to reveal the nature of developmental changes. Second, it is now commonly accepted that development is conjointly driven by structural maturation of the brain as well as skill learning. However, more evidence is needed to understand when and how genes and the environment influence the human brain, especially the differences between brain systems. Moreover, the underlying physiological bases of behavior performances at different ages remain largely unclear. Further studies employing multimodal data should be conducted to ascertain the genetic/environment-brain-behavior model during development. Third, according to previous discussions, several inconsistencies existed in the findings of connectomes in different imaging modalities, and a significantly increased function-and-structure coherence was observed. Although we summarized this as the earlier maturation of structural networks compared with functional networks (Figure 5), further studies are still needed to explore whether these discrepancies reflect additional biological information or limitations of the analysis or imaging methods. Fourth, some new connectome analysis approaches have been raised recently, such as dynamic connectivity, which more comprehensively describes the brain's dynamic integration, coordination and responses to internal and external stimuli across multipletime scales (Hutchison et al., 2013), and network controllability, which reflects the underlying mechanism of brain transformation between cognitive states (Gu et al., 2015b). Further studies using these methods on brain connectome development would provide additional information. Fifth, novel imaging acquisition protocols emerged recently. For example, multiband fMRI (Feinberg et al., 2010; Moeller et al., 2010) with high sampling rates may provide temporally complementary information about functional integration among brain regions and simultaneously reduce the effects of high frequency physiological noise compared with traditional fMRI with low sampling rates (Liao et al., 2013). Further studies with these new protocols will dramatically increase our knowledge of network development.

## Author contributions

MC and YH designed the study; MC, HH and YH wrote this manuscript; YP and QD provided interpretation and revisions of the manuscript.

### Conflict of interest statement

The authors declare that the research was conducted in the absence of any commercial or financial relationships that could be construed as a potential conflict of interest.
